# The HBZ-SP1 isoform of human T-cell leukemia virus type I represses JunB activity by sequestration into nuclear bodies

**DOI:** 10.1186/1742-4690-4-14

**Published:** 2007-02-16

**Authors:** Patrick Hivin, Jihane Basbous, Frédéric Raymond, Daniel Henaff, Charlotte Arpin-André, Véronique Robert-Hebmann, Benoit Barbeau, Jean-Michel Mesnard

**Affiliations:** 1 Laboratoire Infections Rétrovirales et Signalisation Cellulaire, CNRS/UM I UMR 5121/IFR 122, Institut de Biologie, 34000 Montpellier, France; 2 Institut de Génétique Moléculaire, UMR 5535/IFR 122, 1919 Route de Mende, 34293 Montpellier Cedex 5, France; 3 Département des Sciences Biologiques, Université du Québec à Montréal, Montréal, Canada

## Abstract

**Background:**

The human T-cell leukemia virus type I (HTLV-I) basic leucine-zipper factor (HBZ) has previously been shown to modulate transcriptional activity of Jun family members. The presence of a novel isoform of HBZ, termed HBZ-SP1, has recently been characterized in adult T-cell leukemia (ATL) cells and has been found to be associated with intense nuclear spots. In this study, we investigated the role of these nuclear bodies in the regulation of the transcriptional activity of JunB.

**Results:**

Using fluorescence microscopy, we found that the HBZ-SP1 protein localizes to intense dots corresponding to HBZ-NBs and to nucleoli. We analyzed the relative mobility of the EGFP-HBZ-SP1 fusion protein using fluorescence recovery after photobleaching (FRAP) analysis and found that the deletion of the ZIP domain perturbs the association of the HBZ-SP1 protein to the HBZ-NBs. These data suggested that HBZ needs cellular partners, including bZIP factors, to form HBZ-NBs. Indeed, by cotransfection experiments in COS cells, we have found that the bZIP factor JunB is able to target delocalized form of HBZ (deleted in its nuclear localization subdomains) into the HBZ-NBs. We also show that the viral protein is able to entail a redistribution of JunB into the HBZ-NBs. Moreover, by transfecting HeLa cells (known to express high level of JunB) with a vector expressing HBZ-SP1, the sequestration of JunB to the HBZ-NBs inhibited its transcriptional activity. Lastly, we analyzed the nuclear distribution of HBZ-SP1 in the presence of JunD, a Jun family member known to be activated by HBZ. In this case, no NBs were detected and the HBZ-SP1 protein was diffusely distributed throughout the nucleoplasm.

**Conclusion:**

Our results suggest that HBZ-mediated sequestration of JunB to the HBZ-NBs may be causing the repression of JunB activity *in vivo*.

## Background

Human T-cell leukemia virus type I (HTLV-I) is an oncogenic retrovirus etiologically associated with the development of adult T-cell leukemia (ATL) [1,2]. The mechanisms behind leukemogenesis are not yet fully understood but it seems that several events in HTLV-I-infected cells are required for the development of the full malignant phenotype. Among them, the expression of the viral Tax protein plays a crucial role in the first steps of the process [3,4]. Tax has the ability to deregulate the transcription of genes and signaling pathways involved in cellular proliferation, cell cycle control and apoptosis, including deregulation of the activator protein-1 (AP-1), nuclear factor-κB, and E2F pathways [5].

AP-1 represents a dimeric protein, consisting of homodimers and heterodimers composed of basic region-leucine zipper (bZIP) proteins. AP-1 can be formed through either homodimerization of Jun proteins (c-Jun, JunB, and JunD) or heterodimerization of Jun and Fos proteins (c-Fos, FosB, Fra-1, and Fra-2) via their respective ZIP domain [6]. In addition, Jun proteins can heterodimerize with different members of the bZIP protein family including the dimerization partners JDP1 and JDP2 [7], activating transcription factors [8], and Maf proteins [9]. In unstimulated T cells, the basal protein level of AP-1 is low but there is a rapid induction of AP-1 activity after T-cell stimulation. The IL-2 gene was one of the first T-cell-specific genes shown to have an AP-1 site within its promoter [10]. The AP-1 transcription complex has been shown to be involved in the regulation of IL-2 gene expression in combination with other transcription factors [11]. The production of IL-2 by activated T cells is critical for T-cell proliferation and differentiation, and the development of T-cell-dependent immune responses. Over the recent years, a large quantity of data has accumulated demonstrating the contribution of AP-1 to the regulation of numerous cellular genes involved in lymphocyte activation.

AP-1 is also involved in the dysregulated phenotypes of T cells infected with HTLV-I [12,13]. Previous studies have shown that T-cell lines infected by HTLV-I express high levels of AP-1 activity [14,15] with increased levels of mRNAs coding for c-Jun, JunB, JunD, c-Fos, and Fra-1 [16,17]. Indeed, Tax can induce the expression of the genes encoding c-Fos, Fra-1, c-Jun, and JunD [16,18]. In addition, it has been recently demonstrated that Tax enhances AP-1 activity at the post-transcriptional level by activating protein kinase B [19]. Moreover, AP-1-binding sites have been shown to be responsive elements targeted by Tax in different cellular genes such as *fra-1 *and *IL-2 *[20,21]. On the other hand, most of ATL cells do not express significant level of Tax *in vivo *suggesting that constitutive activation of AP-1 in leukemic cells is likely Tax independent [15], although it cannot be completely excluded that a trace amount of Tax may be sufficient for AP-1 activation. However, recent data have suggested the involvement of another viral protein in the regulation of AP-1 activity, *i.e*. the HTLV-I bZIP factor (HBZ) [22].

**Figure 1:**
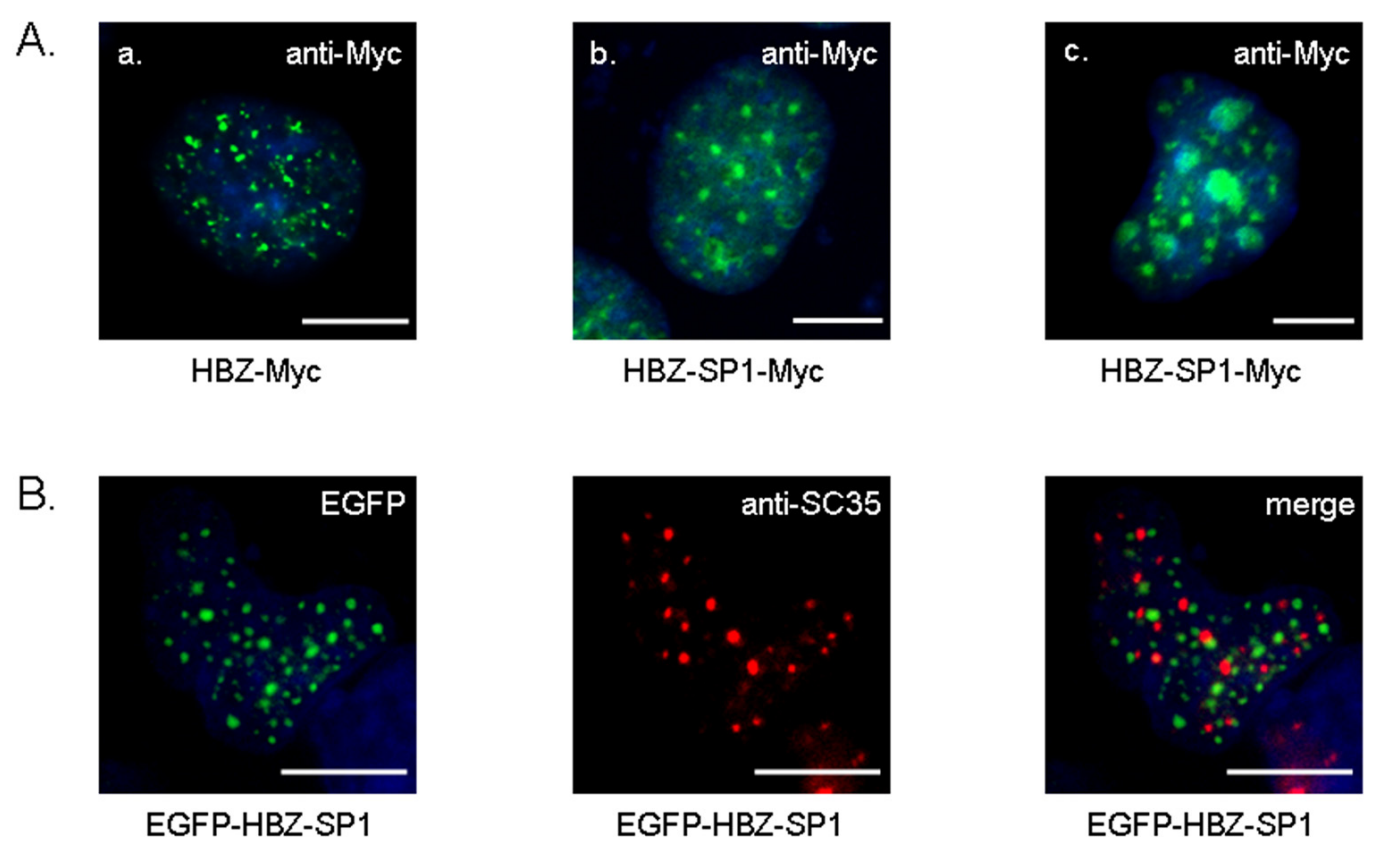
**Subcellular localization of HBZ-SP1**. (A) Subnuclear localization of the HBZ-SP1 protein in transfected COS cells. The original HBZ (a) and HBZ-SP1 (b and c) isoforms fused to the Myc epitope were transiently transfected into COS cells. Cells were cultivated on glass slides, fixed and treated with Vectashield containing DAPI for direct observation by fluorescence microscopy. For immunofluorescence analysis, the anti-Myc antibody was detected with goat anti-mouse IgG antibody coupled to FITC. (B) HBZ-SP1 does not colocalize with endogenous SC35. COS cells transfected with pEGFP-HBZ-SP1 were labelled with a mouse anti-SC35 antibody and detected using goat anti-mouse IgG antibody coupled to Texas Red. Analysis of the green, red, and merged fluorescent signals was performed by fluorescence microscopy. The white bars correspond to a scale of 10 μm.

Unlike Tax, HBZ is encoded by the complementary strand of the HTLV-I genome [23]. Various transcripts initiate from the 3' long terminal repeat (LTR) of the proviral DNA allowing the production of different isoforms of HBZ [24,25]. These isoforms share about 95% amino acid sequence identity and differ only at their N termini. However, the most abundant HBZ form detected in ATL cell lines corresponds to the 206 amino acid-long isoform [24,26] produced from the alternative spliced variant, which we have termed HBZ-SP1 [25]. This messenger RNA can be detected in numerous infected cell lines [25-27] and directly in cells isolated from infected patients [24-26]. The HBZ protein has been described to enhance infectivity and persistence in HTLV-I-inoculated rabbits [28], an observation which might be consequential to the down-regulating ability of HBZ on Tax-dependent viral transcription [23]. HBZ (Fig. 1A) is a prototypical bZIP transcriptional factor [23] with an N-terminal transcriptional activation domain, a central domain involved in nuclear localization, and a C-terminal bZIP domain [29]. HBZ interacts with c-Jun [30,31], JunB [30], and JunD [32] through its bZIP domain. On the other hand, HBZ is unable to interact with c-Fos [31] or to form stable homodimers [30]. The interaction of HBZ with c-Jun leads to a reduction in c-Jun DNA-binding activity [33] and prevents this protein from activating transcription of AP-1-dependent promoters and the HTLV-I promoter (at the basal level) [30]. We have recently demonstrated that the HBZ-SP1 isoform was also able to down-regulate viral expression and to inhibit c-Jun-mediated transcription [25] as already described for the original HBZ isoform.

In this paper, we describe the nuclear distribution of the new HBZ isoform and we show that the HBZ-SP1 protein not only accumulates in particular nuclear bodies (called here HBZ-NBs) as already described for the original HBZ isoform, but that it is also targeted to nucleoli. Moreover, we have studied the *in vivo *nuclear dynamics of the HBZ-NBs through fluorescence recovery after photobleaching (FRAP) experiments on cells transfected with the expression vector encoding HBZ-SP1 fused to the enhanced-green-fluorescent protein (EGFP). We have observed that the rate of nuclear flux of the HBZ-SP1 protein is altered by the deletion of its leucine zipper domain, suggesting that its heterodimerization partners are involved in controlling its own nuclear trafficking. Indeed, by cotransfection experiments in COS cells, we have found that JunB targets a mutated and delocalized form of HBZ into the HBZ-NBs. In addition, we show that HBZ-SP1 also modifies the localization of exogenous JunB in cotransfected COS cells and targets JunB to the HBZ-NBs. Moreover, we demonstrate in HeLa cells (known to have high expression level of JunB) that the relocalization of endogenous JunB by HBZ-SP1 into HBZ-NBs inhibits its transcriptional activity. Taken together, these results clearly demonstrate that HBZ-mediated sequestration of JunB to these particular subnuclear structures may result in repression of JunB activity.

## Results

### The HBZ-SP1 isoform shows a characteristic nuclear distribution

It has recently been shown that the HBZ-SP1 isoform was preferentially expressed in ATL cell lines [24]. For this reason, it was of high interest to investigate the subnuclear distribution of this protein *in vivo*. COS cells were transfected with vectors expressing the original HBZ and HBZ-SP1 isoforms tagged with the Myc epitope fused to its C-terminal end. As shown in (Fig. 1A, a and 1A, b), the subnuclear distribution of the HBZ-SP1 isoform exhibits a NB-associated granular distribution as already described for the original HBZ isoform [29]. We had also shown that this particular nuclear distribution did not correspond to the splicing factor compartments [29]. To determine whether it was also the case for the HBZ-SP1 protein, we next checked the staining pattern of HBZ-SP1 (tagged with EGFP fused to its N-terminus) with that seen in the same cell stained with anti-SC35, an antibody that recognizes one component of an active spliceosome. We found that the HBZ-SP1 isoform did not colocalize with the endogenous SC35 (Fig. 1B).

**Figure 2:**
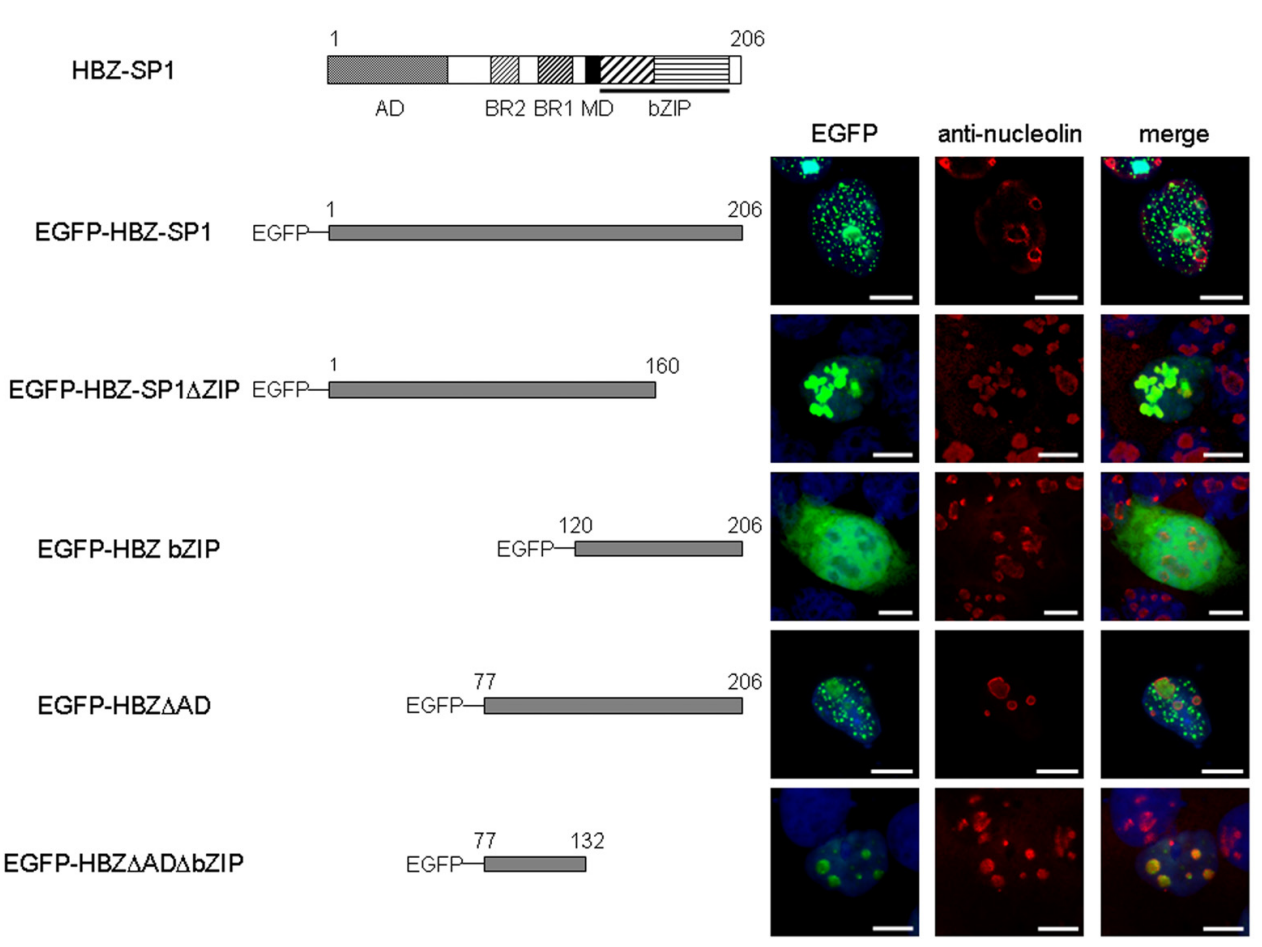
**Molecular structures and nuclear localizations of HBZ-SP1 and its deletion mutants fused to EGFP**. HBZ is composed of an N-terminal activation domain (AD), two basic regions (BR1 and BR2) involved in its nuclear transport, a transcriptional modulatory domain (MD), and a C-terminal bZIP domain. The HBZ-SP1 protein and its mutants fused to EGFP were transiently transfected into COS cells (column EGFP). Cells were cultivated on glass slides, fixed and treated with Vectashield containing DAPI for direct observation by fluorescence microscopy. Transfected COS cells were also labelled with a mouse anti-nucleolin antibody (column anti-nucleolin) and detected using goat anti-mouse IgG antibody coupled to Texas Red. Analysis of the green, red, and merged fluorescent (column merge) signals was performed by fluorescence microscopy. The white bars correspond to a scale of 10 μm.

On the other hand, in the majority of transfected cells, the HBZ-SP1 protein showed a distinct staining pattern when compared to the specific nuclear staining by the original HBZ isoform. Indeed, in addition to the HBZ-NBs, we observed intense spots in the nuclei in structures resembling nucleolus organizing regions (Fig. 1A, c). Colocalization experiments carried out with an anti-nucleolin antibody effectively confirmed that the HBZ-SP1 protein was partly localized to the nucleoli (Fig. 2).

### FRAP analysis of the subnuclear transport of HBZ-SP1

**Figure 3:**
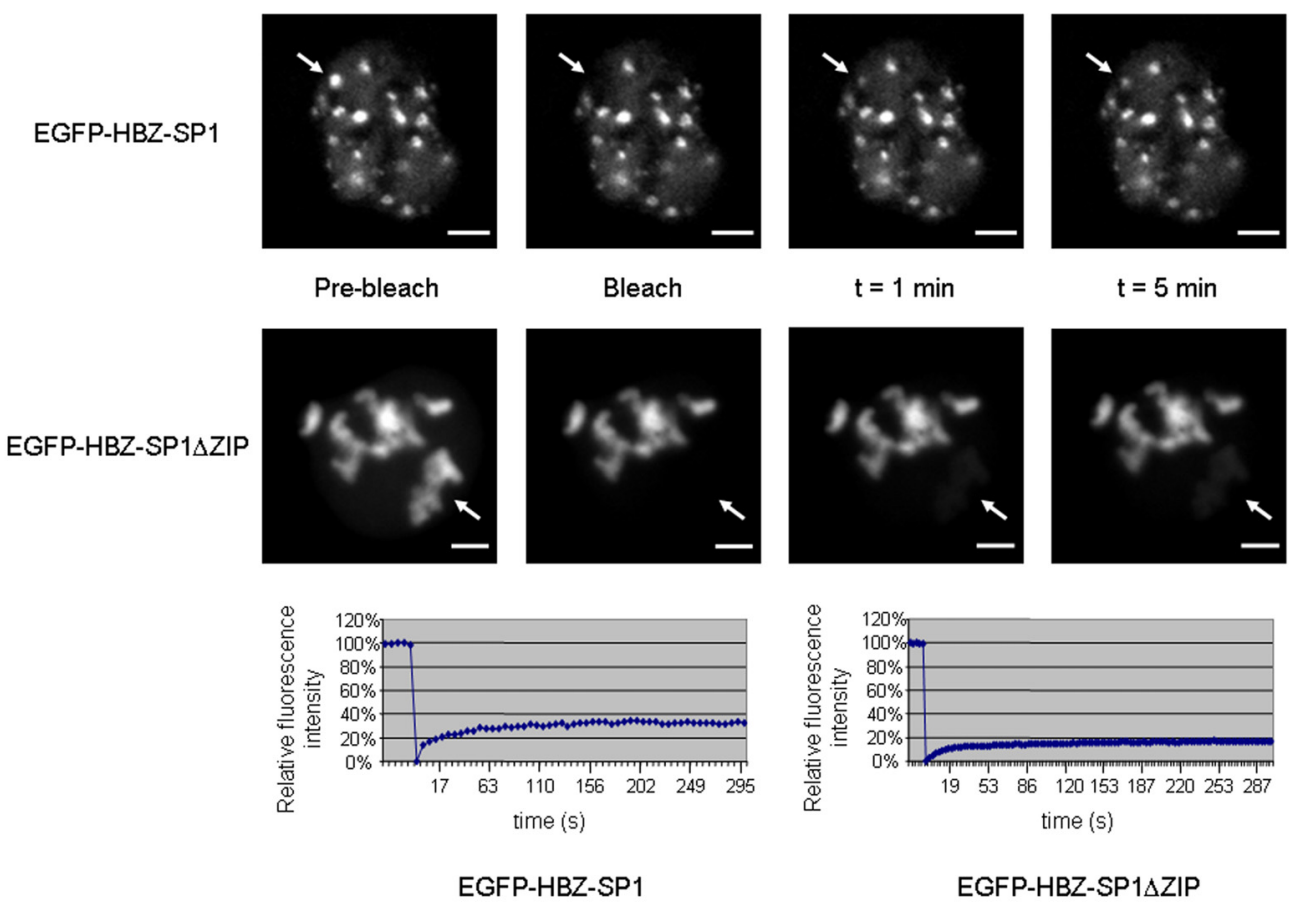
**The HBZ-SP1 protein dynamically associates with subnuclear foci in living cells in a C-terminus-dependent mechanism**. COS cells were transiently transfected with EGFP-HBZ-SP1 or EGFP-HBZ-SP1ΔZIP. Pre-bleached images are shown. The arrows show the photobleached foci for which the recovery rates were determined. The images shown were captured before photobleaching (Pre-bleach) and at the indicated time points (Post-bleach). The white bars correspond to a scale of 10 μm. Recovery curves of the proteins are shown as relative fluorescence intensity vs. time.

The relative intracellular mobility of the EGFP-HBZ-SP1 fusion protein was examined using FRAP analysis. COS cells were transiently transfected with pEGFP-HBZ-SP1 and a defined area in the nucleoplasm of cells expressing the protein was photobleached. Recovery of the fluorescent signal in the entire bleached area was determined by capturing sequential images following photobleaching (Fig. 3). The estimated half-time for signal recovery of EGFP-HBZ-SP1 was 11.5 ± 1.5 s (*n *= 10) and the mean percentage of mobile fraction was 34.0 ± 3.1%. We also observed that the nuclear foci containing EGFP-HBZ-SP1, when recovered after photobleaching, retained the morphology and the nuclear location observed before photobleaching (Fig. 3). These findings were reproduced in three independent experiments. On the other hand, when a defined area in nucleoli containing EGFP-HBZ-SP1 protein was photobleached, the mean percentage of mobile fraction of EGFP-HBZ-SP1 was 90.0 ± 10.0% (data not shown). Moreover, the EGFP control (corresponding to EGFP alone) was observed to be completely mobile (data not shown), confirming that EGFP mobility was modified by its fusion with HBZ-SP1.

To determine the influence of HBZ-SP1 cellular partners on its intracellular mobility, we decided to perform FRAP analysis with EGFP fused to the mutant HBZ-SP1ΔZIP. Interestingly, compared with the wild type, the mutant showed a different pattern of staining since HBZ-SP1ΔZIP-associated structures were more diffuse. As shown in Fig. 2, colocalization experiments carried out with an anti-nucleolin antibody demonstrated that these nuclear structures corresponded to nucleoli displaying a branching structure. These data suggest that the integrity of the viral protein is required for the formation of the HBZ-NBs. Moreover, while the estimated half-time for signal recovery was the same for EGFP-HBZ-SP1 and EGFP-HBZ-SP1ΔZIP, the mean percentage of mobile fraction of EGFP-HBZ-SP1ΔZIP only was 16.4 ± 1.5% (Fig. 3). The decreased mobility of the HBZ-SP1ΔZIP protein compared with the full-length protein also suggested that deletion of the ZIP domain perturbs the intracellular mobility of HBZ-SP1 protein. In conclusion, one plausible interpretation for these observations is that HBZ needs cellular partners, including bZIP factors, to form HBZ-NBs.

### Association of HBZ-SP1 with JunB is involved in the formation of HBZ-NBs

**Figure 4:**
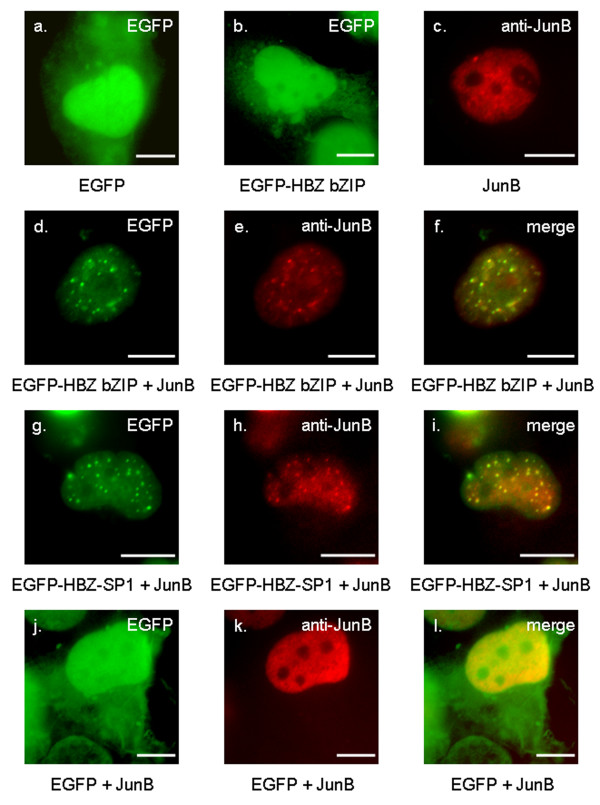
**Stimulation of HBZ-NBs formation by JunB**. COS cells were transiently transfected with EGFP (a), HBZ bZIP fused to EGFP (b), or JunB (c), cultivated on glass slides, fixed, and then were analyzed by fluorescence microscopy. JunB was detected using a mouse anti-JunB antibody and goat anti-mouse IgG antibody coupled to Texas Red. JunB was also cotransfected into COS cells with either EGFP-HBZ bZIP (d-f), or EGFP-HBZ-SP1 (g-i), or EGFP (j-l). Analysis of the green (a, b, d, g, and j), red (c, e, h, and k) and merged (f, i, and l) fluorescent signals was performed by fluorescence microscopy. The white bars correspond to a scale of 10 μm.

To confirm that cellular proteins might be involved in HBZ-NBs formation, we studied the subcellular localization of an HBZ mutant deleted in its N-terminal region while retaining the amino acid sequence from residues 120 to 206 (still containing the C-terminal domain able to interact with bZIP factors). This mutant fused to EGFP (EGFP-HBZ bZIP; Fig. 2) exhibited a staining pattern identical to that of EGFP (compare Fig. 4a and 4b), with a diffuse distribution throughout the cytoplasm and the nucleus. This observation was expected since we have previously shown that HBZ possesses two basic regions BR1 and BR2 (Fig. 2) involved in its nuclear transport located upstream from the bZIP domain but absent in HBZ bZIP [29]. The localization of this mutant was then analyzed in the presence of JunB. This cellular factor was specifically chosen for these analyses since the effect of HBZ on JunB activity still remains unclear. In fact, while HBZ decreases JunB DNA-binding activity *in vitro*, HBZ surprisingly stimulates the collagenase promoter activity in the presence of JunB in CEM cells [30]. However, this activation is very weak and dose-independent suggesting that it could be due to an HBZ-dependent stimulation of an endogenous cellular factor, which binds to the collagenase promoter, *i.e*. JunD [34]. Moreover, JunB showed a diffuse pattern in the nuclei of transfected COS cells, which was easy to discriminate from the HBZ-NB pattern (Fig. 4c). Interestingly, when COS cells were co-transfected with HBZ bZIP and JunB expression vectors, both proteins were modified in their cellular distribution (Fig. 4d–f). Indeed, they colocalized in nuclear spots, which are similar to that observed in the nuclei after cotransfection of COS cells with JunB and the wild type HBZ-SP1 (4g–i). It is worth noting that we have previously described such a staining pattern for JunB in the presence of the original HBZ isoform [30]. In addition, while JunB did not modify the staining pattern of the EGFP control (Fig. 4j–l), the EGFP-HBZ bZIP staining was reduced in the cytoplasm in the presence of JunB (compare Fig. 4b with Fig. 4d). Altogether, these results show that the presence of JunB leads to the nuclear accumulation of HBZ bZIP and that the association of both proteins is involved their targeting into NBs. They also confirm that cellular partners of HBZ are involved in its nuclear trafficking.

**Figure 5:**
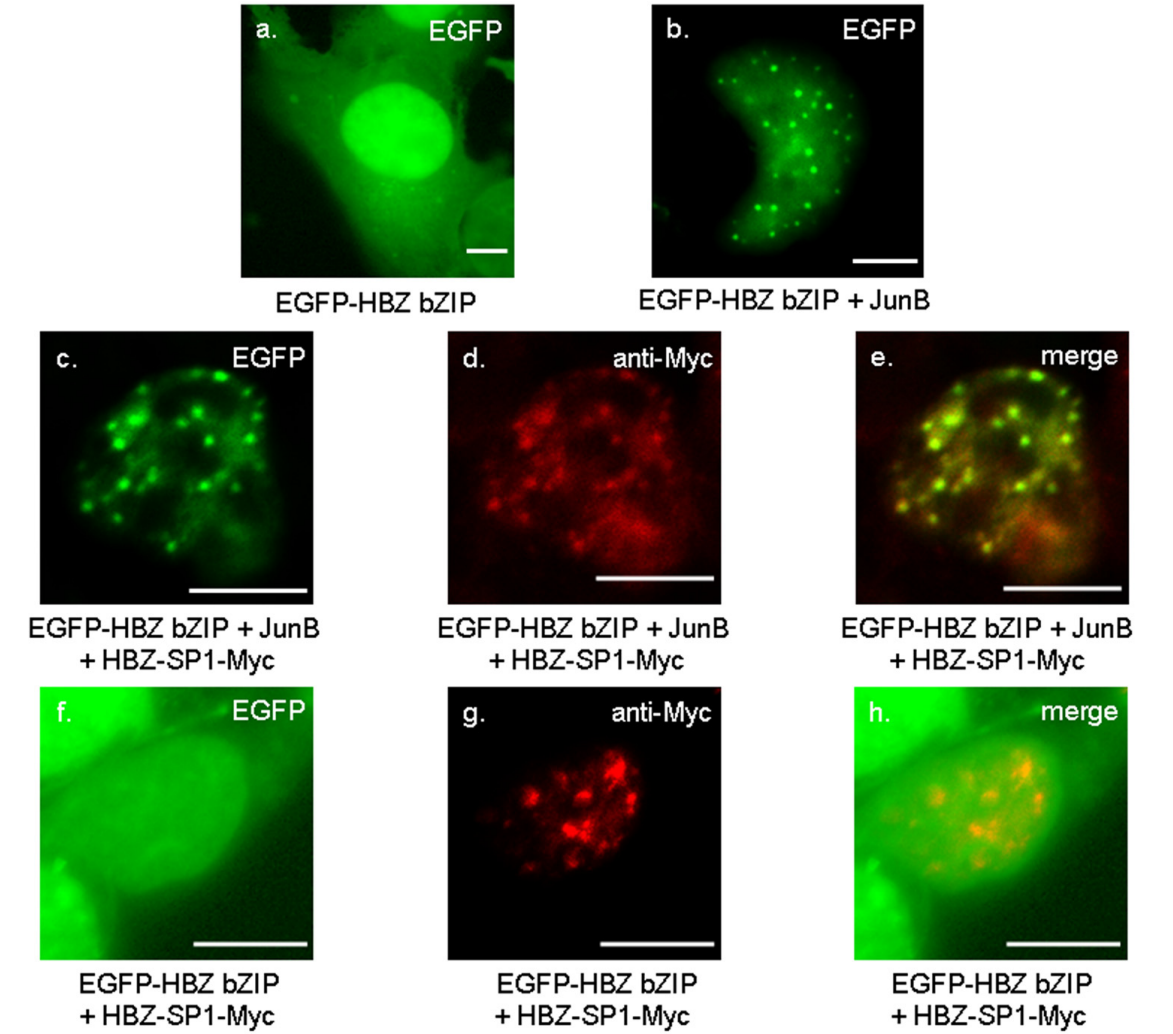
**JunB and HBZ bZIP are involved in the formation of HBZ-NBs**. COS cells were cotransfected with pcDNA-JunB and pEGFP-HBZ bZIP in the absence (b) or in the presence of pcDNA-HBZ-SP1-Myc (c-e). Analysis of the green (a, b, c, and f), red (d and g), and merged (e and h) fluorescent signals was performed by fluorescence microscopy. The HBZ-SP1 protein was detected using the anti-Myc antibody and the goat anti-mouse IgG antibody coupled to Texas Red. COS cells transfected with pEGFP-HBZ bZIP (but without JunB) in the absence (a) or in the presence of pcDNA-HBZ-SP1-Myc (f-h) were also analyzed through the same approach. The white bars correspond to a scale of 10 μm.

To be sure that the nuclear spots formed in the presence of JunB and HBZ bZIP corresponded to HBZ-NBs induced by HBZ-SP1, we analyzed the localization of the different proteins in COS cells transfected with pcDNA-JunB and pEGFP-HBZ bZIP in the absence or in the presence of pcDNA-HBZ-SP1-Myc by fluorescence microscopy. Using this approach, an anti-JunB antibody was not needed to detect JunB. Indeed, the cotransfected cells were easily characterized by the presence of nuclear spots visualized by the green fluorescence due to the targeting of EGFP-HBZ bZIP by JunB into specific NBs (compare Fig. 4b with 4d or Fig. 5a with 5b). On the other hand, in the presence of pcDNA-HBZ-SP1-Myc (Fig 5c–e), HBZ bZIP, JunB, and the HBZ-SP1 protein were found to colocalize to HBZ-NBs as judged by the yellow colour (Fig. 5e), which corresponds to the merging of the green fluorescence of the EGFP-HBZ bZIP (Fig. 5c) and the red staining of the HBZ-SP1 protein (Fig. 5d) detected by indirect immunofluorescence (the mouse anti-Myc antibody is detected with Texas Red-labelled secondary antibodies). As shown in Fig. 5f–h, this colocalization was not be due to the interaction of EGFP-HBZ bZIP with the HBZ-SP1 protein, which was expected given that HBZ is unable to form stable homodimers [30]. Taken together, our results demonstrate that interactions between JunB and the HBZ bZIP domain are involved in the formation of the HBZ-NBs.

### HBZ-NBs are involved in the repression of JunB activity by HBZ-SP1

**Figure 6:**
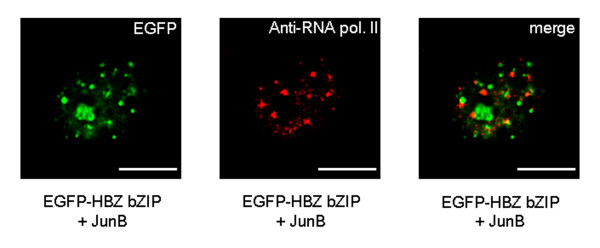
**The HBZ-NBs do not colocalize with endogenous RNA polymerase II**. COS cells cotransfected with pcDNA-JunB and pEGFP-HBZ bZIP were labelled with a mouse anti-RNA polymerase II antibody and detected using goat anti-mouse IgG antibody coupled to Texas Red. Analysis of the green, red, and merged fluorescent signals was performed by fluorescence microscopy. The white bars correspond to a scale of 10 μm.

We have previously suggested that the original HBZ isoform might down-regulate transcription activity of cellular partners by their sequestration in transcriptionally inactive nuclear sites [29]. Therefore, we next conducted a comparison between the staining pattern induced by EGFP-HBZ bZIP, JunB and anti-human RNA polymerase antibody specific for Ser-1801 phosphorylated RNA polymerase II (active form). In cotransfected COS cells, we found that endogenous RNA polymerase II did not colocalize with the HBZ-NBs (Fig. 6). On the other hand, in the absence of the viral protein, we found that JunB colocalized with the active form of RNA polymerase II (data not shown). We also examined the subnuclear localization of the full-length HBZ-SP1 protein not only with RNA polymerase II but also with proteins known to be associated with transcriptional active sites such as Tax, SC35, and the promyelocytic leukemia protein (PML). No colocalization with all these proteins was observed (Fig. 1B and data not shown). Taken together, these results suggest the HBZ-SP1 protein might inhibit JunB activity through its sequestration into HBZ-NBs.

**Figure 7:**
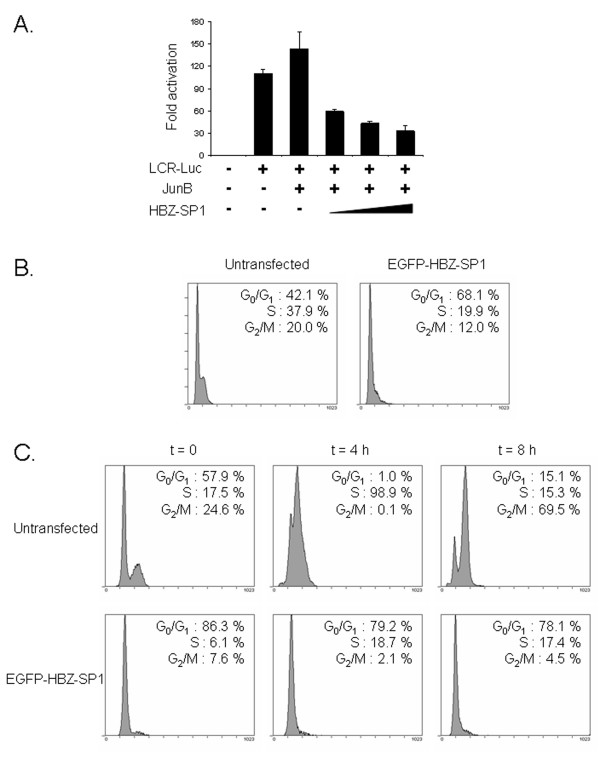
**Effects of the HBZ-SP1 protein on HPV-18 transcription in HeLa cells**. (A) The HBZ-SP1 protein inhibits HPV-18 transcription. HeLa cells (6 × 10^5^) were cotransfected with 0.1 μg of LCR-Luc, 1 μg of pcDNA3.1(-)/Myc-His/*lacZ *(β-galactosidase-containing reference plasmid), 0.5 μg of pcDNA-JunB, and 0, 0.5, 1, or 2 μg of pcDNA-HBZ-SP1-Myc. The luciferase values are expressed as levels of fold activation relative to luciferase activity measured in cells transfected with pcDNA3.1(-)/Myc-His in the presence of the luciferase reporter gene without the early promoter P105. The total amount of DNA in each series of transfection was equal through the addition of pcDNA3.1(-)/Myc-His acting as filler DNA. Luciferase values were normalized for β-galactosidase activity. Values represent the mean ± S.D. (n = 3). (B) HBZ-SP1 protein expression in HeLa cells leads to the accumulation of cells in G_1_. At 24 h posttransfection, cells were harvested and GFP-positive cells (transfected with pEGFP-HBZ-SP1) were then analyzed for DNA content as described in Materials and Methods. The DNA content of EGFP-HBZ-SP1-transfected HeLa cells was then compared with that of untransfected cells by flow cytometry. The experiment shown here is representative of two independent experiments; the other experiment showed similar results. (C) The HBZ-SP1 protein blocks HeLa cell cycle progression through G1 phase. HeLa cells transfected or not with pEGFP-HBZ-SP1 were arrested in the cell cycle using a double thymidine block and restimulated with 10% FCS for the indicated times, harvested, and analyzed as described in panel B. The experiment shown here is representative of three independent experiments; the other two experiments showed similar results.

Human papillomavirus type 18 (HPV-18) mediates HeLa cell proliferation by two oncoproteins, E6 and E7 [35], whose expression is under the control of the early promoter P105 located within the HPV-18 long control region (LCR). Interestingly, it has been demonstrated that P105 is controlled in HeLa cells by an enhanceosome functionally based on a central AP-1 site, which specifically binds the JunB/Fra-2 heterodimer [36]. For this reason, HeLa represented an ideal cell line to study the *in vivo *effects of the HBZ-SP1 protein on JunB transcriptional activity. We first tested the effects of the HBZ-SP1 protein on HPV-18 transcription in the presence of exogenous JunB. HeLa cells were cotransfected with the reporter plasmid pLCR-Luc containing the LCR upstream of the luciferase reporter gene, pcDNA-JunB and increasing amounts of pcDNA-HBZ-SP1-Myc. As shown in Fig. 7A, the stimulation of the luciferase reporter gene by exogenous JunB was weak although this modest induction was likely related to the previously reported high expression level of endogenous JunB in HeLa cells due to a 3-fold amplification of the Jun-B gene [37]. In the presence of the HBZ-SP1 protein, the stimulation of the luciferase reporter gene was inhibited (Fig. 7A). This result was expected since we had already demonstrated that the original HBZ isoform led to a decrease in JunB DNA-binding activity on the AP-1 site [30]. In light of these data, we next investigated whether the HBZ-SP1 protein could affect cell cycle progression of HeLa cells. For these analyses, HeLa cells were transfected with pEGFP-HBZ-SP1 or pNLS-EGFP as a negative control. 24 h after transfection, cells expressing the HBZ-SP1 protein were separated from untransfected cells employing FACS^® ^cell analyzing and both populations were then subjected to cell cycle analysis by measuring DNA content through propidium iodide staining and flow cytometry. The presence of the HBZ-SP1 protein led to the accumulation of cells in G_1 _in contrast to untransfected cells (Fig. 7B). On the other hand, no difference was detected between the cells expressing or not the NLS-EGFP fusion protein (date not shown). Taken together, these results show that the HBZ-SP1 protein is able to down-regulate HPV-18 transcription in HeLa cells and thereby affect cell cycle progression.

**Figure 8:**
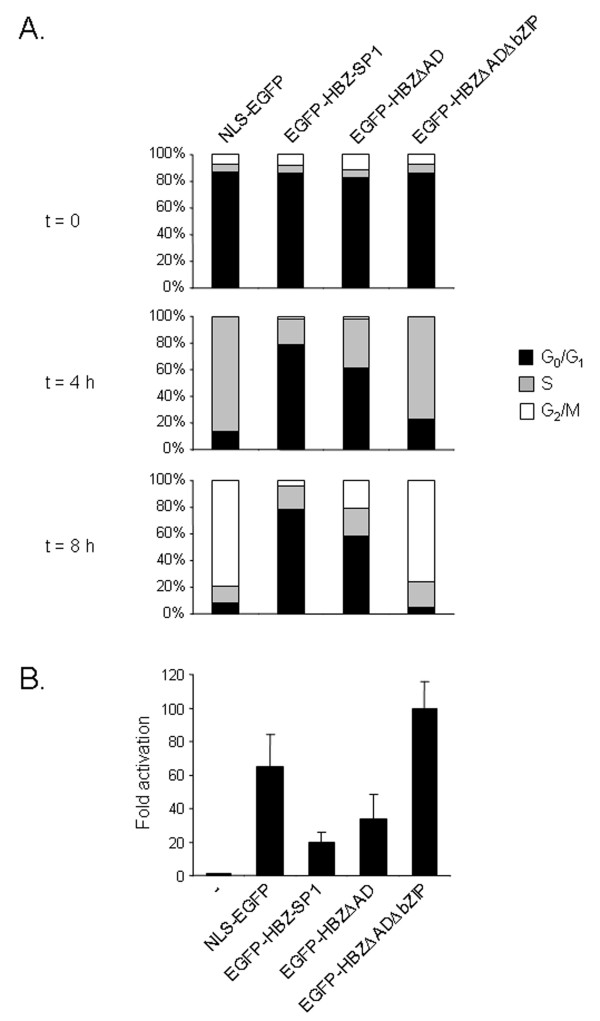
**The bZIP domain of the HBZ-SP1 protein is involved in the cell cycle arrest induced by transfection of HeLa cells with pEGFP-HBZ-SP1**. (A) Cell cycle of HeLa cells transfected with different mutants of the HBZ-SP1 protein. HeLa cells were transfected with pNLS-EGFP, pEGFP-HBZ-SP1, pEGFP-HBZΔAD, or pEGFP-HBZΔADΔbZIP. HeLa cells were arrested using a double thymidine block and restimulated with 10% FCS for the indicated times, harvested, and their DNA content was then analyzed by flow cytometry. Bars show the percentage of cells in each phase of the cell cycle. The data represent results from one of three independent experiments; the other two experiments showed similar results. (B) Effects of EGFP-HBZ-SP1 and the mutants on HPV-18 transcription. HeLa cells were cotransfected with pLCR-Luc and pNLS-EGFP, pEGFP-HBZ-SP1, pEGFP-HBZΔAD, or pEGFP-HBZΔADΔbZIP. The luciferase values are expressed as levels of fold activation relative to luciferase activity measured in cells transfected with pcDNA3.1(-)/Myc-His in the presence of the luciferase reporter gene without the early promoter P105. The total amount of DNA in each series of transfection was equal, through the addition of pEGFP-C2 acting as filler DNA. Luciferase values were normalized for β-galactosidase activity. Values represent the mean ± S.D. (n = 3).

**Figure 9:**
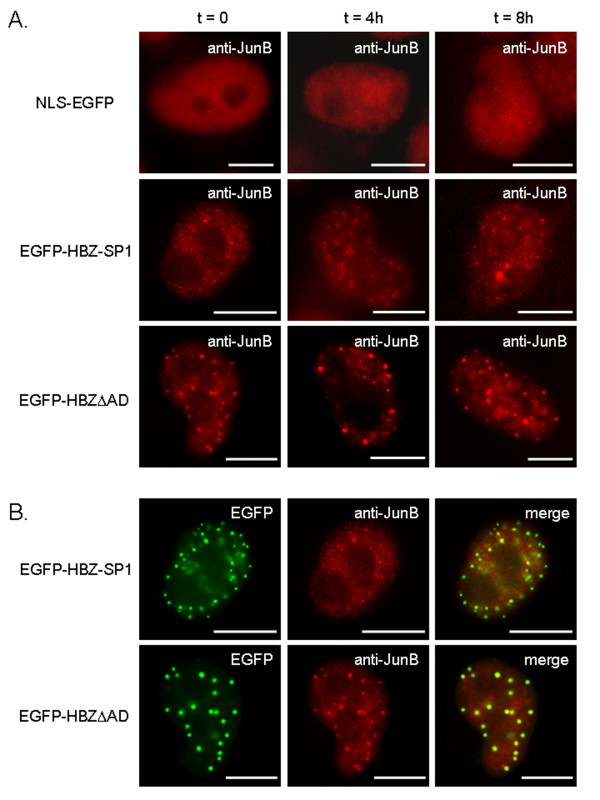
**Targeting of endogenous JunB to HBZ-NBs in HeLa cells transfected with pEGFP-HBZ-SP1**. (A) Nuclear localization of endogenous JunB in HeLa cells transfected with different mutants of the HBZ-SP1 protein. HeLa cells were transfected with pNLS-EGFP, pEGFP-HBZ-SP1, pEGFP-HBZΔAD. HeLa cells were arrested using a double thymidine block and restimulated with 10% FCS for the indicated times and the subnuclear localization of endogenous JunB was analyzed by immunofluorescence microscopy. JunB was detected using a mouse anti-JunB antibody and goat anti-mouse IgG antibody coupled to Texas Red. (B) Colocalization of endogenous JunB with EGFP-HBZ-SP1 and EGFP-HBZΔAD in transfected HeLa cells arrested in the cell cycle. The shown data correspond to t = 0 of Fig. 9A and are representative of the experiments obtained at different times. Analysis of the green, red, and merged fluorescent signals was performed by fluorescence microscopy. The white bars correspond to a scale of 10 μm.

We then analyzed HPV-18 transcription, cell cycle progression, and nuclear distribution of endogenous JunB in HeLa cells transfected with either pEGFP-HBZ-SP1, two mutants (pEGFP-HBZΔAD and pEGFP-HBZΔADΔbZIP; see Fig. 2) or pNLS-EGFP. To better analyze the effects of these different fusion proteins on cell cycle progression, transfected HeLa cells were arrested using a double thymidine block and restimulated by serum to enter the cell cycle. Cell cycle profiles were then analyzed at different time points as described above with the asynchronized HeLa cells. In contrast to untransfected cells, quiescent cells transfected with pEGFP-HBZ-SP1 failed to progress through the G_1_/S transition when they were stimulated by serum to enter the cell cycle, (Fig. 7C). The fusion protein deleted of its activation domain was still able to slow down cell cycle progression since only 20.8% of cells expressing HBZΔAD were in G_2 _phase compared with 79.1% in control cells transfected with pNLS-EGFP (at 8 h) (Fig. 8A). On the other hand, an additional deletion in the C-terminal region of the protein (pEGFP-HBZΔADΔbZIP) completely abrogated the ability of HBZ-SP1 to affect cell cycle progression (Fig. 8A). Interestingly, we found that the EGFP-HBZ-SP1 and EGFP-HBZΔAD fusion proteins were able to negatively regulate P105 promoter activity in HeLa cells, while EGFP-HBZΔADΔbZIP demonstrated no repressing activity on this promoter (Fig. 8B). In parallel, we also analyzed the nuclear distribution of endogenous JunB in transfected HeLa cells, treated with thymidine and restimulated by the addition of serum. In HeLa cells expressing EGFP-HBZ-SP1 or EGFP-HBZΔAD, endogenous JunB was specifically targeted to HBZ-NBs (Fig. 9A) and colocalized with the viral proteins (Fig. 9B). On the other hand, the signal remained diffuse in control cells transfected with pNLS-EGFP (Fig. 9A) or pEGFP-HBZΔADΔbZIP (data not shown). Taken together, our results demonstrate that the targeting of endogenous JunB into HBZ-NBs inhibits JunB activity in HeLa cells.

### The HBZ-SP1 protein does not form HBZ-NBs in the presence of JunD

**Figure 10:**
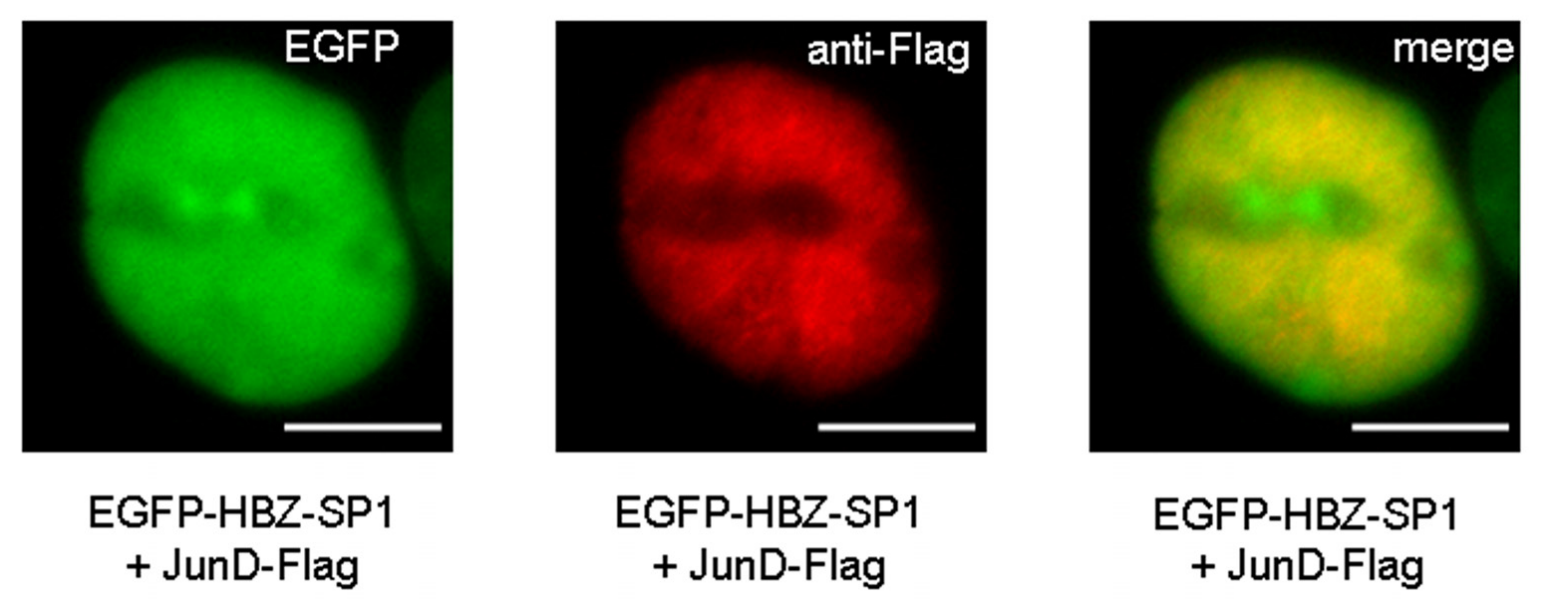
**Immunofluorescence microscopy analysis of the colocalization of JunD and the HBZ-SP1 protein *in vivo***. COS cells cotransfected with pCMV-JunD-Flag and pEGFP-HBZ-SP1 were labelled with a mouse anti-Flag antibody and detected using goat anti-mouse IgG antibody coupled to Texas Red. Analysis of the green, red, and merged fluorescent signals was performed by fluorescence microscopy. The white bars correspond to a scale of 10 μm.

Our data suggest that the HBZ-SP1 protein could inhibit the activity of a transcriptional factor such as JunB by sequestration to HBZ-NBs. HBZ is known to affect transcriptional activity differently depending on its heterodimerization partner. For example, unlike for JunB, HBZ stimulates JunD activity [32]. For this reason, the nuclear distribution of the EGFP-HBZ-SP1 fusion protein was studied in the presence of JunD. COS cells were transiently cotransfected with vectors expressing the EGFP-HBZ-SP1 fusion protein and JunD tagged with the Flag epitope to its C-terminal end. When the JunD expression vector was transfected alone, it localized to the nucleus in a diffuse pattern (data not shown). When EGFP-HBZ-SP1 and JunD were coexpressed, EGFP-HBZ-SP1 was diffusely distributed throughout the nucleus (Fig. 10). The colocalization of JunD and EGFP-HBZ-SP1 in the nucleus was visualized by yellow staining corresponding to the merging of the immunofluorescence signals for both EGFP-HBZ-SP1 and JunD detected. This observation supports the notion that the HBZ-SP1 protein does not target JunD into transcriptionally-inactive NBs.

## Discussion

In this paper, we have studied the nuclear distribution of the HBZ-SP1 isoform produced from the HBZ-SP1 spliced transcript initiated in the 3' long terminal repeat of the HTLV-I proviral genome [24,25]. The HBZ-SP1 mRNA has been described to be the most abundant HBZ spliced variant detected in different HTLV-I-infected cell lines and importantly in cellular clones isolated from HTLV-I-infected patients [24-26]. In addition, by immunoblot analyses and immunochemistry, Murata et al. have recently demonstrated that the ATL cell lines predominantly express HBZ-SP1 isoform at the protein level [24]. They have also observed that the HBZ-SP1 protein can be located in the nucleoli [24] in addition to its association with particular NBs, which have been already described for the original HBZ isoform [23]. Our data here confirm their observations since, in transfected COS cells, the EGFP-HBZ-SP1 fusion protein is effectively located in the nucleoli. Moreover, although we have previously demonstrated that the original HBZ could be associated with heterochromatin distributed around the nucleoli [29], we found no association of the HBZ-SP1 protein with heterochromatin markers (data not shown). The location of the HBZ-SP1 protein in the nucleoli is not unexpected since we have previously demonstrated that HBZ possesses two basic regions, BR1 and BR2, positioned upstream of its bZIP domain, which are similar in sequence to the consensus nucleolar localization signal and are able to target EGFP to nucleoli [29]. The functional role for this particular subcellular targeting remains however unclear. The nucleolus has been described to be involved in the sequestration of transcription factors, such as hypoxia-inducible factor-1α, as well as of proteins that modulate transcription factor activity, such as Mdm2 [38,39]. Therefore, we cannot exclude the possibility that nucleolar proteins can regulate the function of the HBZ-SP1 protein. Conversely, it could be suggested that the HBZ-SP1 protein might act on nucleolar proteins, especially knowing that this viral protein has been shown to be located in nucleoli of ATL cell lines [24].

In addition to BR1 and BR2, a third NLS region corresponding to the basic domain of the HBZ bZIP has been characterized [29]. However, the presence of at least two of the three NLSs is necessary for the translocation of this protein to the nucleus. In this study, we confirm these previous observations since the EGFP-HBZ bZIP fusion protein exhibits a diffuse distribution throughout the cytoplasm and the nucleus. On the other hand, in the presence of JunB, nuclear accumulation of HBZ bZIP is stimulated. Moreover, our results indicate that HBZ bZIP is efficiently targeted into HBZ-NBs by interaction with JunB. On the other hand, this interaction causes targeting of both proteins into NBs and not into the nucleoli, confirming that BR1 and BR2 are likely to be involved in the transport of the viral protein into the nucleoli. In addition, we find that HBZ-NBs do not colocalize with nuclear proteins known to be associated with active transcriptional sites. Effectively, in HeLa cells transfected with EGFP-HBZ-SP1 fusion protein, we have demonstrated that the relocalization of endogenous JunB into HBZ-NBs is associated with repression of its activity. It is worth noting that we have previously demonstrated that the DNA-binding activity of JunB on AP-1 site is inhibited in the presence of HBZ [30]. Taken together, our results suggest that the HBZ-SP1 protein could inhibit JunB activity by forming a heterodimer unable to bind to viral or cellular promoters and by targeting JunB to HBZ-NBs. This hypothesis is confirmed by the data obtained with JunD. Indeed, HBZ stimulates JunD transcriptional activity [32], does not modify its DNA-binding activity [32], and is diffusely distributed throughout the nucleus in the presence of JunD. The latter result differs from a previous study performed in our laboratory. We had found that HBZ entailed an intracellular redistribution of JunD into NBs [32]. We speculate that this difference may be related to the fact that JunD was tagged with RFP, which has impaired the JunD transcriptional activity *in vivo *(our unpublished data). Henceforth, it would be of high interest to determine whether other nuclear proteins could be associated with the HBZ-NBs. For the moment, we have tested different proteins including PML, SC35, HP1α, HP1β, HP1γ, and HMGA1a (data not shown), and none of these proteins demonstrated colocalization with the HBZ-SP1 protein. These particular nuclear structures could also correspond to sites promoting protein degradation as suggested by Matsumoto et al. [31] or protein modification as already described for members of the protein inhibitor of activated STAT family [40]. Complementary experiments are necessary to evaluate these different possibilities.

## Conclusion

We and others have already demonstrated that the HBZ bZIP domain is involved in the interaction with the different members of the Jun family [30,31]. In this study, we further extend these observations with our results on JunB. From our studies, it is apparent that HBZ is capable of inhibiting both c-Jun and JunB transcriptional activities while it stimulates JunD activity. Thus, HBZ provides an interesting model to better understand the consequences of a dysfunction of the AP-1 pathway in T cells. Future experiments focussed on the regulation of HBZ expression and activity will also be of great interest. Moreover, the identification of putative cellular genes controlled by this novel AP-1 factor should elucidate the diverse regulatory properties of HBZ and its exact function in the development of ATL.

## Methods

### Plasmids

The vectors pcDNA-HBZ-SP1-Myc, pEGFP-HBZ-bZIP (Fig. 1A), pcDNA-JunB, pCMV-JunD-Flag have already been described [23,25,30]. To generate the EGFP fusion proteins, HBZ-SP1, HBZ-SP1ΔZIP, HBZΔAD, and HBZΔADΔbZIP DNA (Fig. 1A) were PCR amplified from the pcDNA-HBZ-SP1-Myc vector, digested with *Eco*RI and *Bam*HI, and subcloned in frame into similarly digested pEGFP-C2 (Clontech). pEGFP-HBZΔAD and pEGFP-HBZΔADΔbZIP encode for the HBZ-SP1 protein from residues 77 to 206 and 77 to 132, respectively. pLCR-Luc and pNLS-EGFP (corresponding to EGFP under the control of the nuclear localization signal of SV40) is a gift from G. Steger and M. Piechaczyk, respectively.

### Fluorescence microscopy analysis

COS cells were cultured in DMEM supplemented with 10% FCS. 24 h before transfection, cells were seeded onto glass slides. They were transfected using the jetPEI™ transfection reagent (Qbiogene) according to the manufacturer's instructions and, after 48 h, they were washed with PBS, fixed, and permeabilized with 4% paraformaldehyde and 0.1% Triton ×-100 for 30 min at room temperature. If necessary, cells were incubated with primary antibody (mouse anti-Myc antibody 9E10 or anti-Flag, Sigma-Aldrich; mouse anti-nucleolin, anti-JunB, or anti-RNA polymerase II antibody, Santa Cruz Biotechnology Inc.; the mouse anti-SC35 antibody is a gift from J. Tazi) for 1 h at room temperature. Samples were washed with PBS and then incubated with secondary FITC- or Texas Red-labelled antibodies (Pierce) for 1 h at room temperature. Coverslips were mounted with Vectashield containing DAPI (Abcys) for direct observation.

Fluorescence images were acquired by fluorescence microscopy (model DM R; Leica) at room temperature with a 63x, NA 1.32, oil immersion objective at pinhole size 1 Airy (observation with immersion oil type DF, Cargille Laboratories Inc.). DAPI, GFP, FITC, and Texas Red were excited by 365-, 488-, 492-, and 596-nm laser light and emission was detected at 420, 507, 520, and 620 nm, respectively. The photographs were taken with a Photo Leika DC 250 camera and the images were analyzed with QFluoro software (Leica).

### FRAP

FRAP was performed using the Zeiss LSM Meta 510 confocal microscope. FRAP recoveries were acquired at 37°C on EGFP-HBZ-SP1- or EGFP-HBZ-SP1ΔZIP-expressing cells plated on glass coverslips. The 488-nm line of the Ar+ laser was used for the excitation of EGFP. Cells were observed using a 63x, NA 1.2, oil immersion objective at pinhole size 1 Airy (observation with immersion oil Immersol™, Zeiss). After 5 prebleach scans, a region of interest was bleached and fluorescence recovery was analyzed for 5 min. Experimental recoveries were normalized and corrected for z-position fluctuation of cells using another region as an internal standard.

### HeLa cell transfection and luciferase assay

HeLa cells were cultured in DMEM supplemented with 10% FCS and were transfected using the jetPEI™ transfection reagent (Qbiogene). 1 μg of pcDNA3.1(-)/Myc-His/*lacZ *(β-galactosidase-containing reference plasmid) was included in each transfection to control for transfection efficiency. The total amount of DNA in each transfection was the same (3.6 μg) through the adequate added amount of pcDNA3.1(-)/Myc-His. Cell extracts normalized for protein content were used for luciferase and β-galactosidase assays as already described [30].

### Cell cycle analysis by flow cytometry

Cell cycle analysis was based on the measurement of nuclear DNA content using flow cytometry and propidium iodide. Briefly, HeLa cells were trypsinized, washed with PBS, and fixed in cold 70% ethanol. After being washed in PBS, cells were resuspended in PBS containing 0.1% Triton ×-100, 40 μg/ml propidium iodide, and 50 μg/ml RNase A. Stained cells were then processed with an EPICS XL4C cytofluorometer (Coulter) and analyzed with the CellCycle software. For flow cytometric analysis of transiently transfected cells, 10^6 ^HeLa cells were transfected with 5 μg of pNLS-EGFP or pEGFP-HBZ-SP1 using the BD Calphos™ mammalian transfection kit (BD Biosciences) according to the manufacturer's instructions. At 24 h posttransfection, cells were harvested and GFP-positive cells were then analyzed for DNA content with a UV laser by the EPICS XL4C cytofluorometer (Coulter). For analyses of S-phase entry, the cells were arrested by using a double thymidine block. The HeLa cells were blocked for 12h with 2,5 mM thymidine, washed with DMEM without serum, transfected with the indicated vector and, at 12 h posttransfection, cells were again blocked with 2,5 mM thymidine for another 12 h to arrest all cells at the beginning of S phase. The cells were released from the thymidine block by three washes in fresh medium and allowed to progress through G_1 _and into S phase by stimulation with 10% FCS. The cell cycle positioning of serum-stimulated cells was determined at different time points by propidium iodide staining and flow cytometry analysis as described above.

## Abbreviations

AP-1: activator protein-1

ATL: adult T-cell leukemia

bZIP: basic region-leucine zipper

EGFP: enhanced-green-fluorescent protein

FRAP: fluorescence recovery after photobleaching

HBZ: HTLV-I bZIP

HTLV-I: human T-cell leukaemia virus type I

## Competing interests

The author(s) declare that they have no competing interests.

## Authors' contributions

PH performed the FRAP analyses and most of the fluorescence microscopy analyses, and helped in drafting and finalizing the manuscript. JB and FR have carried out the cell cycle experiments and have been helped by VRH to analyse them by flow cytometry. DH has carried the luciferase assays in HeLa cells and has performed the fluorescence microscopy analysis with JunD. CAA has helped in the construction of the different plasmids. BB and JMM have conceived the study, helped in drafting the manuscript and finalizing the manuscript. All authors read and approved the final manuscript.

## References

[B1] Gallo RC (2005). The discovery of the first human retrovirus: HTLV-1 and HTLV-2. Retrovirology.

[B2] Takatsuki K (2005). Discovery of adult T-cell leukemia. Retrovirology.

[B3] Grassmann R, Aboud M, Jeang KT (2005). Molecular mechanisms of cellular transformation by HTLV-1 Tax. Oncogene.

[B4] Mesnard JM, Devaux C (1999). Multiple control levels of cell proliferation by human T-cell leukemia virus type 1 Tax protein. Virology.

[B5] Mesnard JM (2006). Regulation of viral and cellular transcription by the human T-cell leukemia virus type I Tax oncoprotein. Thébault S,ed Progress in Virus Research.

[B6] Chinenov Y, Kerppola TK (2001). Close encounters of many kinds: Fos-Jun interactions that mediate transcription regulatory specificity. Oncogene.

[B7] Aronheim A, Zandi E, Hennemann H, Elledge SJ, Karin M (1997). Isolation of an AP-1 repressor by a novel method for detecting protein-protein interactions. Mol Cell Biol.

[B8] Van Dam H, Castellazzi M (2001). Distinct roles of Jun:Fos and Jun:ATF dimers in oncogenesis. Oncogene.

[B9] Kerppola TK, Curran T (1994). Maf and Nrl can bind to AP-1 sites and form heterodimers with Fos and Jun. Oncogene.

[B10] Angel P, Imagawa M, Chiu R, Stein B, Imbra RJ, Rahmsdorf HJ, Jonat C, Herrlich P, Karin M (1987). Phorbol ester-inducible genes contain a common cis element recognized by a TPA-modulated trans-acting factor. Cell.

[B11] Jain J, Loh C, Rao A (1995). Transcription regulation of the IL-2 gene. Curr Op Immunol.

[B12] Hivin P, Thébault S, Mesnard JM (2003). Biological role of the bZIP transcription factor superfamily in T-cell transformation induced by human T-cell leukemia virus type I. Recent Res Devel Biol Chem.

[B13] Hall WW, Fujii M (2005). Deregulation of cell-signaling pathways in HTLV-1 infection. Oncogene.

[B14] Iwai K, Mori N, Oie M, Yamamoto N, Fujii M (2001). Human T-cell leukemia virus type 1 Tax protein activates transcription through AP-1 site by inducing DNA binding activity in T cells. Virology.

[B15] Mori N, Fujii M, Iwai K, Ikeda S, Yamasaki Y, Hata T, Yamada Y, Tanaka Y, Tomonaga M, Yamamoto N (2000). Constitutive activation of transcription factor AP-1 in primary adult T-cell leukemia cells. Blood.

[B16] Fujii M, Niki T, Mori T, Matsuda T, Matsui M, Nomura N, Seiki M (1991). HTLV-1 Tax induces expression of various immediate early serum responsive genes. Oncogene.

[B17] Hooper WC, Rudolph DL, Lairmore MD, Lal RB (1991). Constitutive expression of c-Jun and Jun-B in cell lines infected with human T-lymphotropic virus types I and II. Biochem Biophys Res Commun.

[B18] Fujii M, Sassone-Corsi P, Verma IM (1988). c-fos promoter trans-activation by the tax1 protein of human T-cell leukemia virus type I. Proc Natl Acad Sci USA.

[B19] Peloponese JM, Jeang KT (2006). Role for Akt/protein kinase B and AP-1 in cellular proliferation induced by the human T-cell leukemia virus type 1 (HTLV-1) Tax oncoprotein. J Biol Chem.

[B20] Curtiss VE, Smilde R, McGuire KL (1996). Requirements for interleukin 2 promoter transactivation by the Tax protein of human T-cell leukemia virus type 1. Mol Cell Biol.

[B21] Tsuchiya H, Fujii M, Niki T, Tokuhara M, Matsui M, Seiki M (1993). Human T-cell leukemia virus type 1 Tax activates transcription of the human fra-1 gene through multiple cis elements responsive to transmembrane signals. J Virol.

[B22] Mesnard JM, Barbeau B, Devaux C (2006). HBZ, a new important player in the mystery of Adult-T- cell leukemia. Blood.

[B23] Gaudray G, Gachon F, Basbous J, Biard-Piechaczyk M, Devaux C, Mesnard JM (2002). The complementary strand of HTLV-1 RNA genome encodes a bZIP transcription factor that down-regulates the viral transcription. J Virol.

[B24] Murata K, Hayashibara T, Sugahara K, Uemura A, Yamaguchi T, Harasawa H, Hasegawa H, Tsuruda K, Okazaki T, Koji T, Miyanishi T, Yamada Y, Kamihira S (2006). A novel alternative splicing isoform of human T-cell leukemia virus type 1 bZIP factor (HBZ-SI) targets distinct subnuclear localization. J Virol.

[B25] Cavanagh MH, Landry S, Audet B, Arpin-Andre C, Hivin P, Paré ME, Thete J, Wattel E, Marriott SJ, Mesnard JM, Barbeau B (2006). HTLV-I antisense transcripts initiating in the 3'LTR are alternatively spliced and polyadenylated. Retrovirology.

[B26] Satou Y, Yasunaga JI, Yoshida M, Matsuoka M (2006). HTLV-I basic leucine zipper factor gene mRNA supports proliferation of adult T cell leukemia cells. Proc Natl Acad Sci U S A.

[B27] Larocca D, Chao LA, Seto MH, Brunck TK (1989). Human T-cell leukemia virus minus strand transcription in infected T-cells. Bioch Biophys Res Comm.

[B28] Arnold J, Yamamoto B, Li M, Phipps AJ, Younis I, Lairmore MD, Green PL (2006). Enhancement of infectivity and persistence in vivo by HBZ, a natural antisense coded protein of HTLV-1. Blood.

[B29] Hivin P, Frédéric M, Arpin-André C, Basbous J, Gay B, Thébault S, Mesnard JM (2005). Nuclear localization of HTLV-I bZIP factor (HBZ) is mediated by three distinct motifs. J Cell Science.

[B30] Basbous J, Arpin C, Gaudray G, Piechaczyk M, Devaux C, Mesnard JM (2003). HBZ factor of HTLV-I dimerizes with transcription factors JunB and c-Jun and modulates their transcriptional activity. J Biol Chem.

[B31] Matsumoto J, Ohshima T, Isono O, Shimotohno K (2005). HTLV-1 HBZ suppresses AP-1 activity by impairing both the DNA-binding ability and the stability of c-Jun protein.. Oncogene.

[B32] Thébault S, Basbous J, Hivin P, Devaux C, Mesnard JM (2004). HBZ interacts with JunD and stimulates its transcriptional activity. FEBS lett.

[B33] Hivin P, Arpin-André C, Clerc I, Barbeau B, Mesnard JM (2006). A modified version of a Fos-associated cluster in HBZ affects Jun transcriptional potency. Nucleic Acids Res.

[B34] Selvamurugan N, Kwok S, Partridge NC (2004). Smad3 interacts with JunB and Cbfa1/Runx2 for transforming growth factor-1-stimulated collagenase-3 expression in human breast cancer cells. J Biol Chem.

[B35] DeFilippis RA, Goodwin EC, Wu L, DiMaio D (2003). Endogenous human papillomavirus E6 and E7 proteins differentially regulate proliferation, senescence, and apoptosis in HeLa cervical carcinoma cells. J Virol.

[B36] Bouallaga I, Massicard S, Yaniv M, Thierry F (2000). An enhanceosome containing the JunB/Fra-2 heterodimer and the HMG-I(Y) architectural protein controls HPV 18 transcription. EMBO reports.

[B37] Choo KB, Huang CJ, Chen CM, Han CP, Au LC (1995). Jun-B oncogene aberrations in cervical cancer cell lines. Cancer Letters.

[B38] Fatyol K, Szalay AA (2001). The p14ARF tumor suppressor protein facilitates nucleolar sequestration of hypoxia-inducible factor-1a (HIF-1a) and inhibits HIF-1-mediated transcription. J Biol Chem.

[B39] Carmo-Fonseca M (2002). The contribution of nuclear compartmentalization to gene regulation. Cell.

[B40] Shuai K, Liu B (2005). Regulation of gene-activation pathways by PIAS proteins in the immune system. Nature Reviews.

